# Developing (Quantitative Structure Property Relationships) QSPR Techniques to Predict the Char Formation of Polybenzoxazines

**DOI:** 10.3390/polym8050166

**Published:** 2016-04-25

**Authors:** Maryam Sairi, Brendan Howlin, Ian Hamerton

**Affiliations:** 1 Department of Chemistry, Faculty of Engineering and Physical Sciences, University of Surrey, Guildford, Surrey, GU2 7XH, UK; m.sairi@surrey.ac.uk; 2 The Advanced Composites Centre for Innovation and Science, Department of Aerospace Engineering, University of Bristol, Queen’s Building, University Walk, Bristol, BS8 1TR, UK; i.hamerton@bristol.ac.uk

**Keywords:** polybenzoxazines, thermal stability, char formation, mathematical modelling, QSPR

## Abstract

This study uses the Molecular Operating Environment software (MOE) to generate models to calculate the char yield of polybenzoxazines (PBz). A series of benzoxazine (Bz) monomers were constructed to which a variety of parameters relating to the structure (e.g., water accessible surface, negative van der Waals surface area and hydrophobic volume, *etc*.) were obtained and a quantitative structure property relationships (QSPR) model was generated. The model was used to generate data for new Bz monomers with desired properties and a comparison was made of predictions based on the QSPR model with the experimental data. This study shows the quality of predictive models and confirms how useful computational screening is prior to synthesis.

## 1. Introduction

**Scheme 1:**
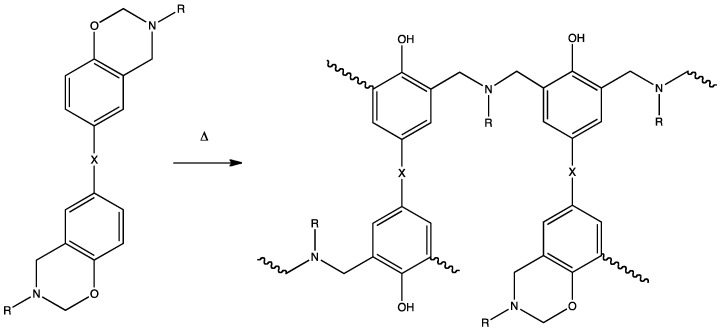
Schematic showing polymerisation of bisbenzoxazines through ring opening and crosslinking.

Thermoset polymers have an established history in civil aviation, in applications involving decorative panels, secondary composite structures and adhesives typically around 90% of the interior furnishings of a typical civil airliner will contain thermoset composites [1]. The development of structural materials with improved thermal stability and fire resistance is key in this area to retard the spread of fire, and modern legislation is leading to the removal of halogenated flame retardants [2]. This is often achieved by introducing highly aromatic or hetero-aromatic materials such as polybenzoxazines (PBZs) [3] (Scheme 1) that form intumescent chars during the combustion process, with the polymer swelling and becoming porous to protect the underlying structure [4]. PBZs are a comparatively recent addition to the commercial thermosetting resins, but there is great interest in their potential as replacements for phenolics [5] or epoxy resins [6] and, whilst they are not currently widely used in civil aviation, they are being evaluated in this application.

Cured PBZs offer a combination of favourable thermal and mechanical performance (e.g., dry *T*_g_ values of 255 °C, wet *T*_g_ = 196 °C are possible [7], coupled with very low moisture uptake) that gives an attractive property profile. PBZs have the potential to compete with conventional phenolics in terms of high thermal stability and flame resistance. In previous work [8] we have examined the thermal stability of cured PBZs and investigated the influence of particle size and the structure of the bisphenyl unit on the manner in which the crosslinked polymer undergoes degradation. Molecular modelling of polymers is a growing area and was reviewed in a special edition of the Journal of Polymer Science in 2015 where Ginzberg, Weinhold and Trefonas stated that “In the near future, modeling is expected to be an integral part of formulation design and the screening process” [9]. It was reported that the modelling work was proven to be useful to predict properties such as the temperature, decomposition, softening and failure of composites on a bulk scale, reflecting the size of some actual components [10]. The work shows the ability of the model to give a good agreement between the prediction data and the experimental data on most of the properties that were examined. While the work was focusing on composites on a bulk scale, we, on the other hand, are trying to utilize modelling on to the molecular and atomistic scale. The whole area of molecular scale modelling of thermosets was reviewed by Li and Strachan where we were credited with publishing the first fully atomistic molecular dynamics simulation of a thermoset [11]. Another way to use atomistic modelling of thermosets is in Quantitative Structure Property Relationships (QSPR) which is the polymer analogue of Quantitative Structure Activity Relationships (QSAR) widely used in drug design to develop new pharmaceuticals. With QSPR we seek to relate the structure of the monomer of particular polymer to the physical, mechanical and thermal properties of the derived polymer using mathematical methods. This technique has been pioneered by Hopfinger [12], Katrizky [13] and Bicerano [14] in particular and is the source of several commercial software packages. We are particularly interested in the potential to predict structure–property relationships and have had some success in using quantitative structure property relationships (QSPR) towards the prediction of e.g., the glass transition temperature or degree of cure achieved [15]. In the current study, we concentrate on the refinement of this method and achieve a level of accuracy that is comparable with the experimental determination of char yield by thermogravimetric analysis (TGA).

## 2. Methodology

Molecular Operating Environment (MOE) software by Chemical Computing Group (Cambridge, UK) was used to run QSPR and generate models to calculate the predicted char yield of thirty-two benzoxazines (the training set). The Partial Least Squares (PLS) regression algorithm was used to analyse the relationship between the actual char yield (measured by experimental work) and the predicted char yield (calculated using the model). PLS was chosen because it contains the fewest number of factors therefore it provides maximum correlation with the dependant variables.

There are six main steps to generate a model with the best final linear model equation:

1. The training data set was chosen from the Handbook of Benzoxazine Resins. This training set is a secondary data set and consists of thirty-two benzoxazines with corresponding actual char yield measured by different research groups.
2. All monomers were built using the builder menu in MOE and a conformational search using Low Mode Molecular Dynamics [16] was carried out on each monomer before energy minimising the lowest energy conformer of each model to convergence.
3. A series of descriptors [17] were calculated for each monomer, which cover molecular volume, shape, charge, *etc*.
4. A QSPR equation as developed to relate the descriptors to the experimentally determined char yield using partial least squares (PLS) [18].
5. Descriptors which play a major role in influencing the model were chosen. The linear model equation with the highest coefficient of determination (*r*^2^) was selected and further analysis was done on this model.
6. The descriptors were then used to calculate the prediction values and the average percentage error of the data produced was calculated *in-silico*.
7. The Leave-One-Out-Cross-Validation test [19] was carried out by the model to evaluate whether it could be taken further and capable to produce accurate prediction values. This test was done by taking out one of the materials in the training set and applying the model to that chosen material.
8. The experimental data of the material used in the validation test was compared against the predicted/calculated data. The percentage error and difference error between the two values was calculated and a conclusion was made based on the comparison values.

## 3. Results and Discussion

The training data set that was used in this research was compiled from various papers in the Handbook of Benzoxazine Resins [3]. The data set consists of thirty-two benzoxazine monomers and their recorded percentage char yields as polybenzoxazines reported by various sources. Char yields are normally taken from Thermogravimetric Analysis (TGA) under a nitrogen atmosphere, as these materials tend to burn away completely in oxygen atmospheres in the TGA. The list of the monomers in the data set is shown in Table 1.

**Figure 1:**
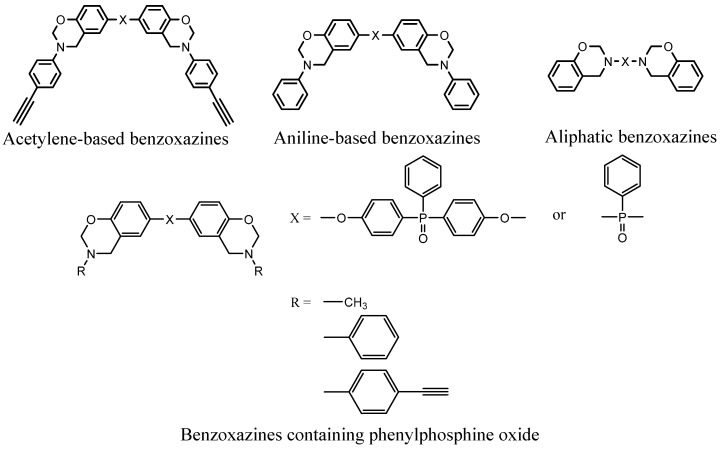
The general structure of some of benzoxazines in the training set.

The training set consists of a mixture various structures of benzoxazines including acetylene-based benzoxazines, aniline-based benzoxazines, aliphatic benzoxazines, benzoxazines containing phenylphosphine oxide, monofunctional benzoxazines and benzoxazines with fused-ring bridges (Figure 1). The full structures of each material are given in the Supplementary Material.

Since the data set is secondary data from a variety of sources, it is expected to contain significant errors as it was reported by different teams from various places, using potentially different methods. This fact was supported by a set of compiled data from the literature that shows the errors from secondary data measurements can be up to 14% (Table 2). For instance, the data set (Table 2) consists of five measurements of the percentage char yield on the polybenzoxazine formed from bisphenol A and aniline (BA-a) reported by different research groups and collected from different articles from the literature.

Based on Table 2, the error associated with the data set is 14%, which exceeds the acceptable 10% experimental error by 4%. However, it is believed that the significantly high error in the measurements is due to the different parameters that were used in the measurements (e.g., different temperature and different heating rate). There are also other potential parameters that might contribute to the large errors in the measurements such as different sample size used, the different thickness and shape of the crucibles and the physical condition of the sample, either in bulk or in powder [27].

As the degrees of freedom were reduced, it was found that the error in the measurements was also reduced. This statement is supported by the data set in Table 3, which combines experimental data for the percentage char yield of the same material, BA-a measured at 800 °C with an experimental heating rate of 10 K/min. It shows that by keeping these two parameters constant, the experimental error was greatly reduced from 14% to only 10.48%.

Table 2 and Table 3 contain examples of secondary data. To compare the quality of secondary data to primary data, a series of measurements are made on the same material and reported by a “single” group (within this department) (Table 4). All parameters were kept constant as much as possible; the same temperature (800 °C), heating rate (10 K/min) and method, including the experimental apparatus. It was found that the measurement readings are very consistent with a very small experimental error which is only 2.26% compared to 10.48% from the previous data set (Table 3).

Since secondary data was used as the training set for the current project, it is therefore to be expected that the percentage error in this work will be of the order of 10% to 14%. The best equation derived for the char yield is shown in Table 5.

The two descriptors with the two highest relative importance ratings are the Oprea Rotatable Bond Count (opr_nrot) with 100% importance and the Number of rotatable bonds (b_rotN) with 75% importance (69% importance from the previous model). The least important descriptor is the logP(o/w) with 0.03 importance. Hence the most important feature of a benzoxazine to increase char yield is the number of rotatable bonds in the monomer.

The prediction data in Table 6 were produced *in-silico* using descriptors from the table above. The errors between the prediction data and the actual data were then calculated manually using Microsoft Excel.

**Figure 2:**
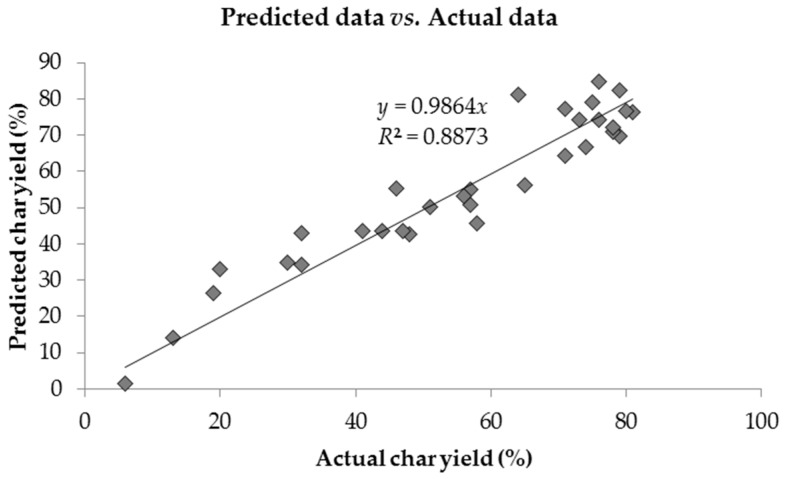
Partial Least Squares (PLS) regression graph of prediction of char yield *vs.* actual char yield of lowest energy molecular structures.

The *R*^2^ value generated is 88.73%. The R^2^ value is above 90% and this shows that there is a reasonable correlation between the actual char yields and the predicted char yields, although the value of *R*^2^ is not as good as hoped (at least 95%).The average error and the average percentage error for this model were found to be 5.77 and 14.58%. A graph of the predicted *versus* actual data is shown in Figure 2.

**Figure 3:**
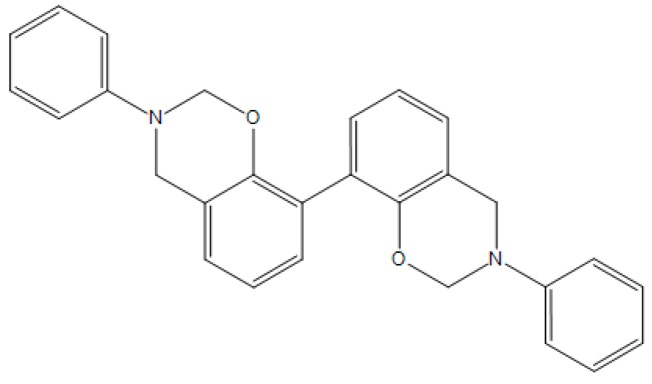
Structure of 22P-a.

The leave-one-out-cross-validation (LOOCV) test was used to validate the model generated. 22P-a was chosen as the one to be left out for the test and the structure of 22P-a is shown in Figure 3.

The actual percentage yield for this material is 45% [26] and the prediction data generated by the linear model equation carried out *in-silico* is 45.13%. The percentage error and the difference error of both readings are less than 1 (Table 7).

The small value of percentage error between the readings shows that although the *r*^2^ is less than 95% and the average percentage error is 13%, the model can still give a good prediction for the chosen material. This is a very interesting as the validation confirmed that the model is capable of predicting the percentage char yields of benzoxazines with common structures. However, the model is not yet powerful enough to carry out a prediction on a benzoxazine with an unfamiliar functional group.

**Figure 4:**
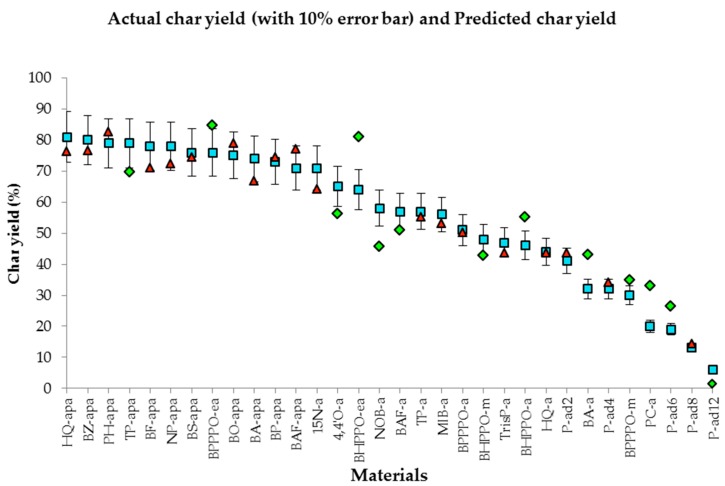
Actual char yield with 10% error bars and prediction char yield plotted together. (Blue squares: Actual char yield; Red triangles: Predicted char yield (within 10% percentage error); Green diamonds: Predicted char yield (exceeds 10% percentage error).

To investigate the model further, a graph of actual char yield (with 10% error bar) and predicted char yield was plotted in Figure 4. A percentage error of 10% was taken as a reference as generally experimental error will fall within this 10% error. Figure 4 shows that there are thirteen benzoxazines whose predicted values exceed the 10% error bars. The list of the molecules and their structures are presented in Table 8.

## 4. Van Krevelen Calculations

The Van Krevelen method [35] doesn’t have group contributions for groups containing sulphur, oxygen and nitrogen atoms that are similar to the structure of the benzoxazine monomers. It also does not include the contribution of halogen atoms to the calculation and we have two benzoxazines with halogen atoms in our data set. We have tried the Van Krevelen prediction method on our benzoxazine set to see if this method will produce a better prediction. However, the result shows that it does not work well with the benzoxazines in our data set with an *R*^2^ of only 59.23% compared to the *R*^2^ produced by our method which is 88.73%.

## 5. Conclusions

The field of QSPR of benzoxazines is developing rapidly, assisted by the compilation of data in accessible reference sources. As with all data, curation is required, particularly with data that does not have a strictly defined value, e.g., glass transition temperature, which—not being a first order thermodynamic transition—can exhibit a range of values. However, data that has a lower degree of “error” is capable of being predicted to within experimental error or to within 10% of the value, e.g., char yield, as shown by this work. However, as with all predictions based on molecular structure, the need for accurate models is paramount and as shown here it is wise to take conformational flexibility into account in the models used. In common with all QSPR modelling, when the structure being modelled is 'unusual' in some way, it leads to a larger error in the predictions. However, with the advent of increasing computer power and accuracy in molecular modelling and the rise of faster data processing, the field will see rapid progress in future. The prediction clearly shows that in order to design benzoxazine monomers that will have a higher char yield then increasing the number of rotatable bonds in the monomer and/or increasing the accessible surface area are valid routes.

## Figures and Tables

**Table 1:** List of % char yield of thirty-three benzoxazine monomers (the training set). The definition of the abbreviation used for each benzoxazine are given in the Abbreviations at the end of the paper.

No.	Materials	Char yield (%)	No.	Materials	Char yield (%)
1	HQ-apa	81 [20]	17	TP-a	57 [21]
2	BZ-apa	80 [20]	18	MIB-a	56 [22]
3	PH-apa	79 [3], (81) [20]	19	BPPPO-a	51 [23]
4	TP-apa	79 [20]	20	BHPPO-m	48 [23]
5	BF-apa	78 [20]	21	TrisP-a	47 [21]
6	BS-apa	78 [20]	22	BHPPO-a	46 [3], (41) [23]
7	NP-apa	76 [20]	23	HQ-a	44 [21]
8	BPPPO-ea	76 [23]	24	P-ad2	41 [24]
9	BO-apa	75 [20]	25	P-ad4	32 [24]
10	BA-apa	74 [20]	26	BA-a	32 [20,25]
11	BP-apa	73 [20]	27	BPPPO-m	30 [23]
12	BAF-apa	71 [20]	28	PC-a	20 [21]
13	15N-a	71 [21]	29	P-ad6	19 [24]
14	4,4'O-a	65 [26]	30	P-ad8	1 [24]
15	BHPPO-ea	64 [23]	31	P-ad12	6 [24]
16	BAF-a	57 [3]	32	NOB-a	58 [22]

**Table 2:** Experimental error of six BA-a char yield measurements conducted at different temperatures and heating rates.

Temperature (°C)	Heating rate (K/min)	Measured char yield (%), Y_c_	Mean, Ȳ_c_	Difference error, Y_c_ − Ȳ_c_	Average difference error	Percentage error (%)	Average percentage error (%)
800	20	24.30 [28]	28.72	4.42	4.06	18.19	14.00
	20	32.00 [20]		3.28		10.25	
	10	35.60 [29]		6.88		19.33	
	10	25.70 [30]		3.02		11.75	
900	20	26.00 [31]		2.72		10.46	

**Table 3:** Experimental error of six BA-a char yield measurements reported at the same temperature (800 °C) and heating rate (10 K/min).

Temperature (°C)	Measured char yield (%), Y_c_	Mean, Ȳ_c_	Difference error, Y_c_ − Ȳ_c_	Average difference error	Percentage error (%)	Average percentage error (%)
800	26.62 [32]	27.78	1.16	3.12	4.36	10.48
	25.00 [33]		2.78		11.12	
	26.00 [34]		1.78		6.85	
	35.60 [29]		7.82		21.97	
	25.70 [30]		2.08		8.09	

**Table 4:** Experimental error of three BA-a char yield measurements done by a “single” group study.

Temperature (°C)	Measured char yield (%), Y_c_	Mean, Ȳ_c_	Difference error, Y_c_ − Ȳ_c_	Average difference error	Percentage error (%)	Average percentage error (%)
800	26.62 [32]	25.87	0.75	0.58	2.80	2.26
	25.00 [33]		0.87		3.49	
	26.00 [34]		0.13		0.49	

**Table 5:** The linear model equation produced from the model and the relative importance of each descriptor.

Percentage char yield = −139.65 (+8.92 × b_rotN) (−7.00× lip_violation) (+1.15 × logP(o/w)) (−10.78 × opr_nrot) (−0.44 × PEOE_VSA-2) (−0.35 × PEOE_VSA-3) (+118.61 × petitjeanSC) (+174.65 × Q_VSA_FNEG) (+0.65 × SMR_VSA6) (−4.82 × std_dim2)		
**Relative importance**	**Descriptors’ abbreviations**	**Description**
1.00	opr_nrot	Oprea Rotatable Bond Count
0.75	b_rotN	Number of rotatable bonds
0.61	SMR_VSA6	Bin 6 SMR (0.485, 0.560)
0.56	Q_VSA_FNEG	Fractional negative vdw surface area
0.34	PEOE_VSA-2	Total negative 2 Å^2^ vdw surface area
0.24	petitjeanSC	(diameter − radius)/radius
0.19	PEOE_VSA-3	Total negative 3 Å^2^ vdw surface area
0.18	lip_violation	Lipinski Violation Count
0.09	std_dim2	Standard dimension 2 Å
0.08	logP(o/w)	Log octanol/water partition coefficient

**Table 6:** Prediction data, calculated average error and average percentage error for lowest energy conformation quantitative structure property relationships (QSPR) model.

Materials	Actual data (%)	Predicted data (%)	Difference error	Average error	% error	Average % error	*R*^2^
HQ-apa	81.00	74.66	6.34	5.02	7.82	12.54	91.94%
BZ-apa	80.00	82.75	2.75		3.44		
PH-apa	79.00	84.69	5.69		7.20		
TP-apa	79.00	75.25	3.75		4.75		
BF-apa	78.00	77.82	0.18		0.23		
BS-apa	76.00	76.55	0.55		0.72		
NP-apa	78.00	82.14	4.14		5.31		
BPPPO-ea	76.00	72.19	3.81		5.02		
BO-apa	75.00	78.04	3.04		4.05		
BA-apa	74.00	64.07	9.93		13.42		
BP-apa	73.00	69.12	3.88		5.31		
BAF-apa	71.00	75.61	4.61		6.49		
15N-a	71.00	65.94	5.06		7.12		
4,4'O-a	65.00	57.64	7.36		11.32		
BHPPO-ea	64.00	73.97	9.97		15.57		
NOB-a	58.00	43.99	14.01		24.15		
BAF-a	57.00	52.39	4.61		8.08		
TP-a	57.00	55.74	1.26		2.22		
MIB-a	56.00	51.58	4.42		7.89		
BPPPO-a	51.00	52.74	1.74		3.41		
BHPPO-m	48.00	41.58	6.42		13.37		
TrisP-a	47.00	40.97	6.03		12.82		
BHPPO-a	46.00	52.51	6.51		14.16		
HQ-a	44.00	45.46	1.46		3.32		
P-ad2	41.00	45.44	4.44		10.83		
BA-a	32.00	45.22	13.22		41.31		
P-ad4	32.00	32.35	0.35		1.09		
BPPPO-m	30.00	34.51	4.51		15.05		
PC-a	20.00	30.69	10.69		53.43		
P-ad6	19.00	25.31	6.31		33.20		
P-ad8	13.00	13.43	0.43		3.31		
P-ad12	6.00	2.65	3.35		55.84		

**Table 7:** Validation value and percentage error.

Material	Actual data (%)	Prediction data (%)	% error	Difference error
22P-a	45.00	45.31	0.69	0.31

**Table 8:** List of benzoxazines with percentage error more than 10%.

Materials	Structures	Materials	Structures
BA-apa	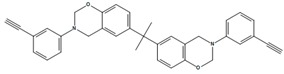	P-ad6	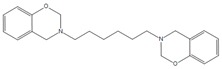
4,4′O-a	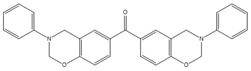	P-ad12	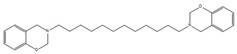
BAF-a	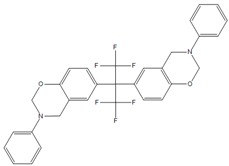	NOB-a	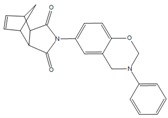
BA-a	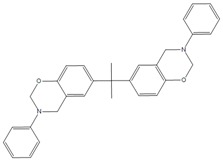	PC-a	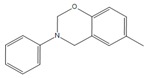
BHPPO-m	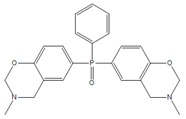	BHPPO-a	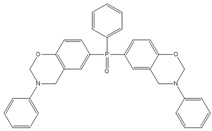
BHPPO-ea	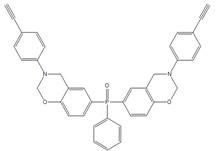	BPPPO-m	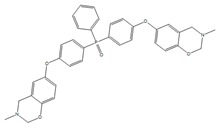
BPPPO-ea	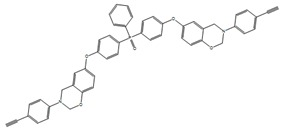		

## Supplementary Materials

The following are available online at www.mdpi.com/2073-4360/8/5/166/s1. Table S1: Structures of benzoxazines in the training set.

## Abbreviations

The following abbreviations are used in this manuscript:

BAF-a: bisphenolAF/aniline benzoxazine benzoxazine
PH-apa: *N*-aminophenyl-acetylene phenol benzoxazine
BF-apa: *N*-aminophenyl-acetylene bisphenol F benzoxazine
HQ-apa: *N*-aminophenyl-acetylene hydroquinone benzoxazine
BA-apa: *N*-aminophenyl-acetylene bisphenol A benzoxazine
BP-apa: *N*-aminophenyl-acetylene 4,4′-dihydroxy biphenyl benzoxazine
TP-apa: *N*-aminophenyl-acetylene 4,4′-thiodiphenol benzoxazine
BAF-apa: *N*-aminophenyl-acetylene bisphenol AF benzoxazine
BS-apa: *N*-aminophenyl-acetylene bisphenol S benzoxazine
BO-apa: *N*-aminophenyl-acetylene bisphenol O benzoxazine
BZ-apa: *N*-aminophenyl-acetylene 4,4′-dihydroxybenzophenone benzoxazine
NP-apa: *N*-aminophenyl-acetylene 2,7-dihydroxynaphtalene benzoxazine
PC-a: *p*-cresol/aniline benzoxazine
BA-a: bisphenol A/aniline benzoxazine
HQ-a: hydroquinone/aniline benzoxazine
15N-a: 1,5-dihydronaphtalene/aniline benzoxazine
TP-a: 4,4′-thiodiphenol/aniline benzoxazine
TrisP-a: 1,1,1-*tris*(*p*-hydroxyphenyl)-ethane/aniline benzoxazine
22P-a: 2,2′-dihydroxybiphenyl/aniline benzoxazine
4,4′O-a: 4,4′-dihydroxybenzophenone/aniline benzoxazine
P-ad2: ethlenediamine bisphenol benzoxazine
P-ad4: *N*-1,4-diaminobutane bisphenol benzoxazine
P-ad6: *N*-1,6-diaminohexane bisphenol benzoxazine
P-ad8: *N*-1,8-diaminooctane bisphenol benzoxazine
P-ad12: *N*-1,12-diaminododecane bisphenol benzoxazine
MIB-a: 1-(4-hydro-phenyl)-pyrrole-2,5-dione/aniline benzoxazine
NOB-a: *p*-hydroxyphenylnadimide/aniline benzoxazine
BHPPO-a: *bis*-(4-hydroxyphenyl)phenylphospine oxide benzoxazine
BHPPO-m: methylamine *bis*-(4-hydroxyphenyl)phenylphospine oxide benzoxazine
BHPPO-ea: 3-ethylaniline *bis*-(4-hydroxyphenyl)phenylphospine oxide benzoxazine
BPPPO-a: *bis*-(4-benzyloxyphenoxy-4′-phenyl)phenyl phosphine/aniline benzoxazine
BPPPO-m: methylamine *bis*-(4-benzyloxyphenoxy-4′-phenyl)phenyl phosphine benzoxazine
BPPPO-ea: 3-ethylaniline *bis*-(4-benzyloxyphenoxy-4′-phenyl)phenyl phosphine benzoxazine
