# Supplementary material for: Developing (Quantitative Structure Property Relationships) QSPR Techniques to Predict the Char Formation of Polybenzoxazines
Source: Polymers (Basel). 2016 Apr 25;8(5):166. doi: 10.3390/polym8050166 (PMC6431917; doi:10.3390/polym8050166)
Supplement: Supplementary file 1 [file polymers-08-00166-s001.pdf]

Supplementary Material: Developing (Quantitative Structure Property Relationship) QSPR Techniques to Predict the Char Formation of Polybenzoxazines

Maryam Sairi, Brendan Howlin and Ian Hamerton

Table S1. Structures of benzoxazines in the training set.

No.	Materials	Structures
1.	HQ-apa	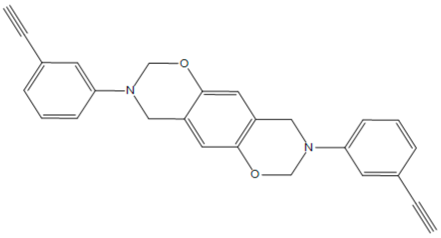
2.	BZ-apa	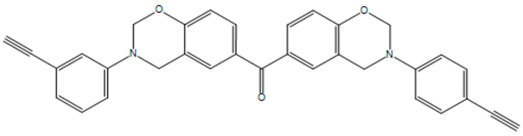
3.	PH-apa	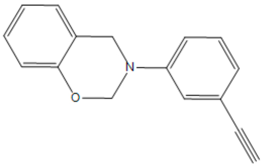
4.	TP-apa	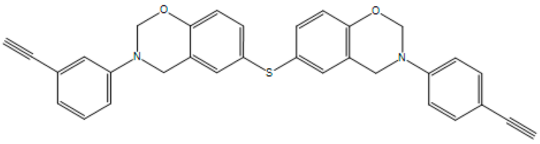
5.	BF-apa	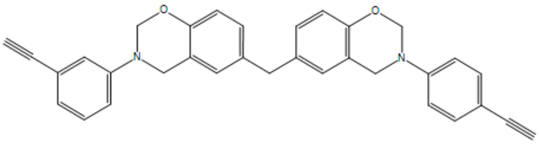
6.	BS-apa	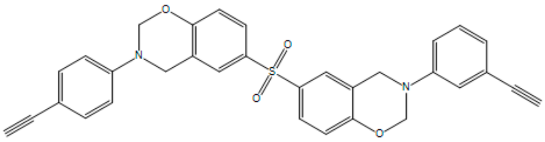
7.	NP-apa	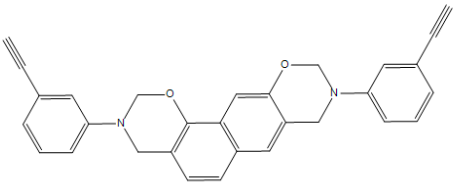

Table S1. *Cont.*

8.	BPPPO-ea	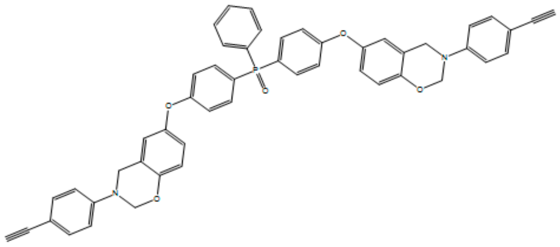
9.	BO-apa	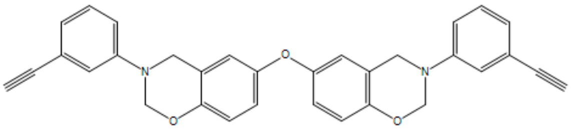
10.	BA-apa	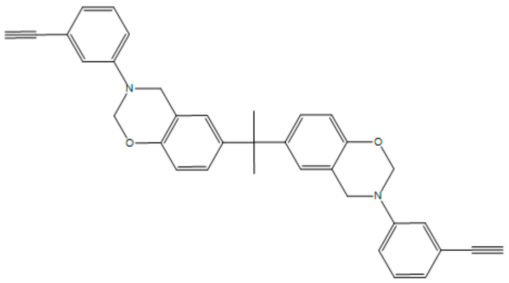
11.	BP-apa	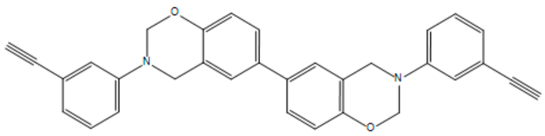
12.	BAF-apa	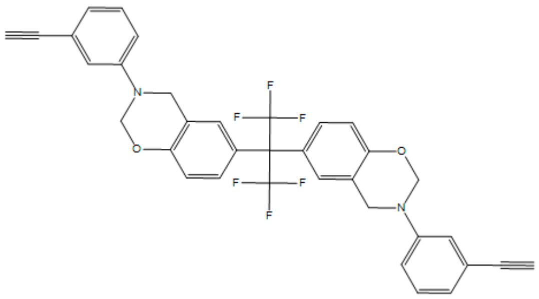
13.	15N-a	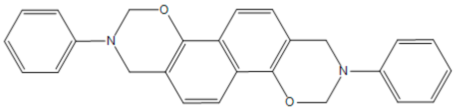
14.	4,4'O-a	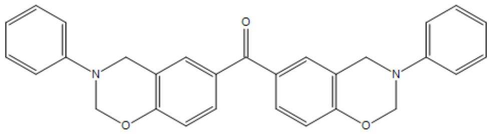

Table S1. *Cont.*

15.	BHPPO-ea	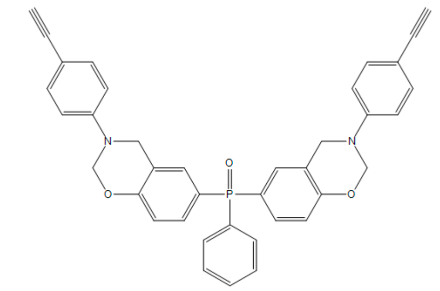
16.	BAF-a	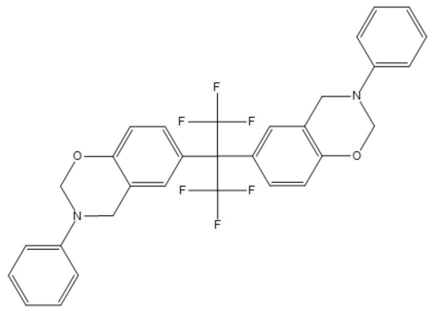
17.	TP-a	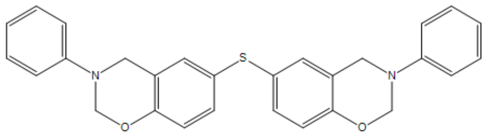
18.	MIB-a	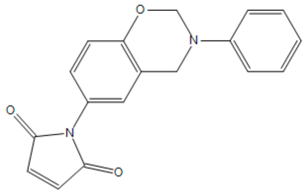
19.	BPPPO-a	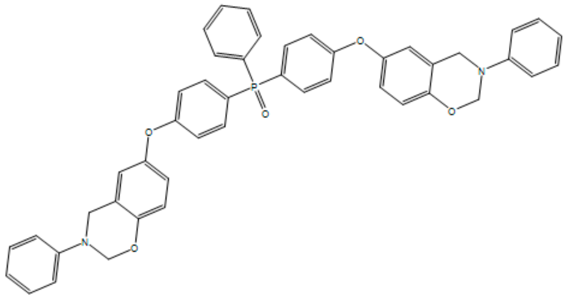
20.	BHPPO-m	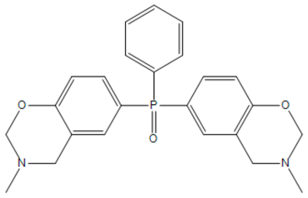

Table S1. *Cont.*

21.	TrisP-a	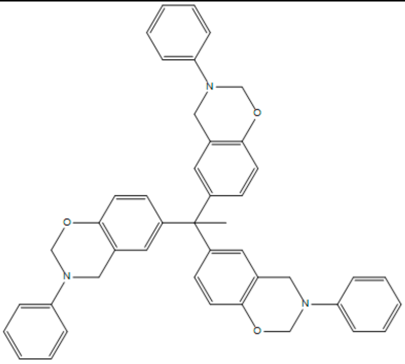
22.	BHPPO-a	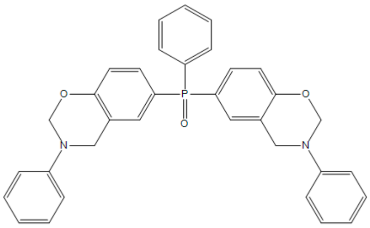
23.	HQ-a	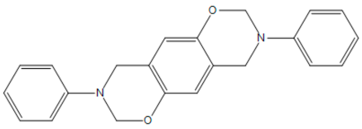
24.	P-ad2	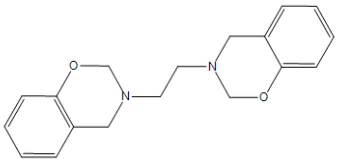
25.	P-ad4	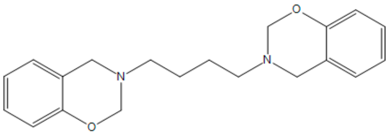
26.	BA-a	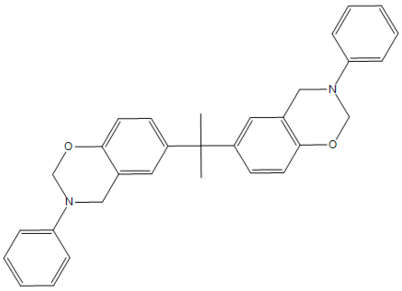
27.	BPPPO-m	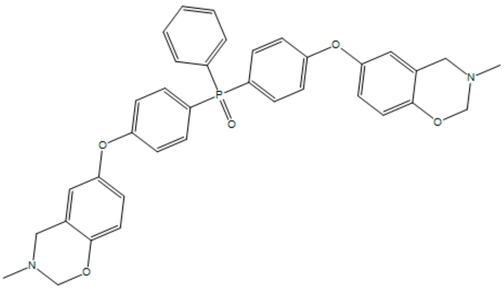

Table S1. *Cont.*

28.	PC-a	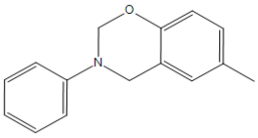
29.	P-ad6	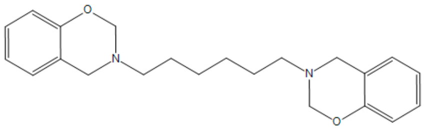
30.	P-ad8	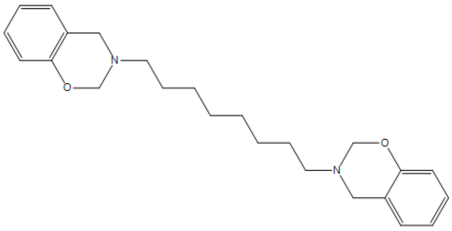
31.	P-ad12	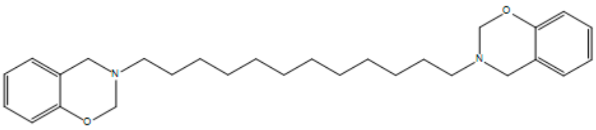
32.	NOB-a	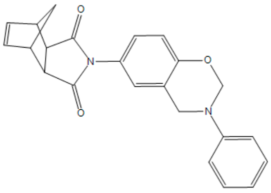

© 2016 by the authors; licensee MDPI, Basel, Switzerland. This article is an open access article distributed under the terms and conditions of the Creative Commons Attribution (CC-BY) license (http://creativecommons.org/licenses/by/4.0/).
